# Contrast enhancement of vascular walls of intracranial high flow malformations in black blood MRI indicates high inflammatory activity

**DOI:** 10.1186/s41016-018-0120-0

**Published:** 2018-05-25

**Authors:** Athanasios K. Petridis, Maxine Dibue-Adjei, Jan F. Cornelius, Marian Preetham Suresh, Lan Li, Marcel A. Kamp, Yousef Abusabha, Bernd Turowski, Hans Jakob Steiger, Rebecca May

**Affiliations:** 1 Department of Neurosurgery, Heinrich Heine University, Duesseldorf, Germany; 2 LivaNova Deutschland GmbH (a LivaNova PLC-owned subsidiary), Lindberghstr 25, D-80939 Munich, Germany; 3 Diagnostic and Interventional Neuroradiology,Heinrich Heine University, Duesseldorf, Germany; 4 Department of Neurosurgery, Niederrhein Hospital, Duisburg, Germany

**Keywords:** Arteriovenous malformation, Arteriovenous fistula, Black blood MRI, Inflammation

## Abstract

**Background:**

There are controversies concerning the natural history of arteriovenous malformations (AVMs) in literature and it is not clear which AVMs should be treated and which should be just observed. Objective criteria beyond growth in serial MRIs or angiographies are needed. The use of black blood MRI is currently under investigation for evaluating the rupture risk of cerebral aneurysms, however its use for assessment of AVMs has yet to be evaluated. We therefore conducted a feasibility study on the application of black blood MRI (bbMRI) in AVMs to assess rupture risk.

**Methods:**

Retrospective study of 10 patients with intracranial AVMs and 4 patients with arteriovenous fistulas who received a black blood MRI before treatment.

**Results:**

AVM niduses (9/10) show contrast enhancement irrespective of rupture or size. All arteriovenous fistulas (4 / 4) were contrast enhancing irrespective of rupture.

**Conclusion:**

High flow malformations are in a permanent stage of inflammation which does not seem to allow conclusions on their rupture risk at the current stage. BbMRI is a feasible method of identifying inflammation in AVMs and arteriovenous fistulas. However, future prospective studies are needed to evaluate whether bbMRI contrast enhancement correlates with rupture risk.

## Background

The natural history of arteriovenous malformations (AVMs) represents a controversial topic. While the ARUBA study shows a low rupture risk, epidemiologic studies suggest a high risk of bleeding throughout a patient´s life [1, 2]. Little is known about the pathophysiological processes in an AVM and even less about any factors which could predict a risk of bleeding. The black blood MRI (bbMRI) appeared on the diagnostic horizon and offers a promising outlook in predicting rupture risk in intracranial aneurysms [3, 4]. As contrast enhancement in the aneurysm wall reflects inflammatory processes and inflammation in the AVM vessel wall has been shown histologically [5], we studied the possibility of bbMRI on high flow AVMs in order to evaluate differences in inflammatory processes which could signify instability. One study with 4 patients using ferumoxytol-enhanced MRI showed the feasibility of MRIs in visualizing inflammation in AVMs [6]. A study on cavernomatous malformations conducted by our group showed no significant contrast enhancement of the walls of brain cavernomas (low flow malformations, results submitted for publication). The main question was whether inflammatory reactions in the walls of high flow malformations can be visualized by bbMRI and whether the contrast enhancement can predict any status of instability of an AVM.

## Methods

From January 2017 until July 2017 patients with AVMs treated in our department received a bbMRI sequence in addition to the regular MRI. We retrospectively analyzed the MRIs of 10 patients with AVMs treated in our department. In addition, *N* = 4 patients with arteriovenous fistulas received a bbMRI. The patients presented in our outpatient facilities or in case of AVM bleeding in the emergency room. The analysis of bbMRIs was performed by a neuroradiologists and the result of contrast enhancement in the AVM was rated as positive or negative. Three cases are illustrated. AVM size was defined as the longest diameter in mm.

Cranial MRI was performed on a 3T MR scanner (Magnetom Skyra, Siemens, Erlangen) with a 20 channel head coil. The protocol included a 3 D T1 space sequence with fat saturation (SPAIR) and blood suppression (field of view 179*230, repetition time 693 ms, echo time 18 ms, matrix 256 x 256, spatial resolution 0.9 x .09 x 0.9 mm) before and after administration of gadolinium (0,2 ml/kg/BW, maximum 20 ml; ProHance, Bracco Imaging, Germany). Total scan time was 8 min.

## Results

*N* = 10 patients with AVMs of which 4 were female received a bbMRI. The mean age of patients with bleeding AVMs (*N* = 4) was 42 y (19-77 y) and 52.8 y (46-61 y) for the non-bleeding cases. Five patients were operated on; 3 AVMs are still under observation, one was embolized and one irradiated. In 9 / 10 cases the AVM vessel walls showed contrast enhancement. Four patients presented with intracranial bleeding all of which showed strong contrast enhancement in their AVM niduses. Only one AVM with no haemorrhage and a Spetzler-Martin Grade 2 had no contrast enhancement. There was no difference in contrast enhancement between bleeding and non-bleeding AVMs. Additionally, the contrast enhancement was localized to the whole nidus, whereas in the 4 studied arteriovenous fistula patients the contrast enhancement was exclusively in the walls of the abnormal vein. All 4 patients with arteriovenous fistulas had contrast enhancement (2 with haemorrhage and 2 without). Further analysis of the patients with fistulous malformation was not possible due to the low sample size.

Table 1 summarizes some of the characteristics of the AVMs in the 10 studied patients.

**Table 1:** Characteristics of the study population

Gender	Age	S-M	Localization	Hemorrhage	Size	Treatment	Contrast enhancement in black blood MRI
Male	51 y	1	temporal	non bleeding	25 mm	Surgery	Contrast at fistulas
Female	19 y	1	temporal	bleeding	15 mm	Surgery	Contrast atnidus
Male	19 y	2	frontal	bleeding	14 mm	Surgery	Contrast at nidus
Male	53 y	2	frontal	bleeding	15 mm	Surgery	Contrast at nidus
Male	77 y	2	cerebellar	bleeding	17 mm	Embolisation	Contrast at nidus
Male	46 y	2	frontal	non bleeding	9 mm	Radiation	No contrast
Female	49 y	2	parietal	non bleeding	36 mm	Observation	Contrast at nidus
Female	53 y	2	temporal	non bleeding	33 mm	Surgery	Contrast at nidus
Female	61 y	4	Temporo-parietal	non bleeding	46 mm	Observation	Contrast at fistulas
Male	57 y	5	fronto-parietal	non bleeding	67 mm	Observation	Contrast at nidus

### Illustrative Cases

We show the typical bbMRI contrast enrichment in the AVMs in the following 3 cases.

**Fig. 1:**
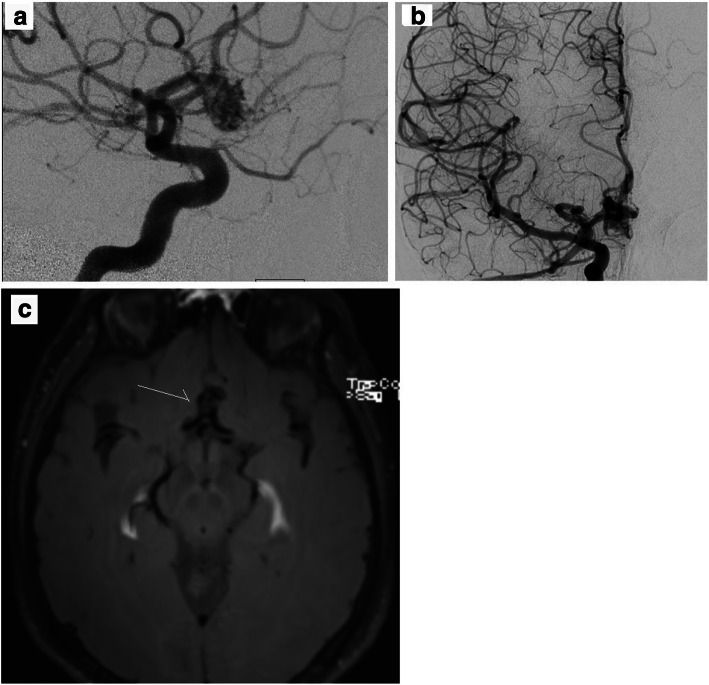
*Case with incidental AVM.* **a** Digital subtraction angiography showing the frontal AVM in sagittal (left) and **b**. coronal (right) views. **c**. BbMRI shows no contrast enhancement in the AVM

The first case illustrates a 46 y.o. male patient who presented in our outpatient clinic with the diagnosis of an incidental AVM of 9 mm and a deep venous drainage. The bbMRI shows no contrast enhancement. Nevertheless, the patient was treated by radiation therapy (Fig. 1).

**Fig. 2:**
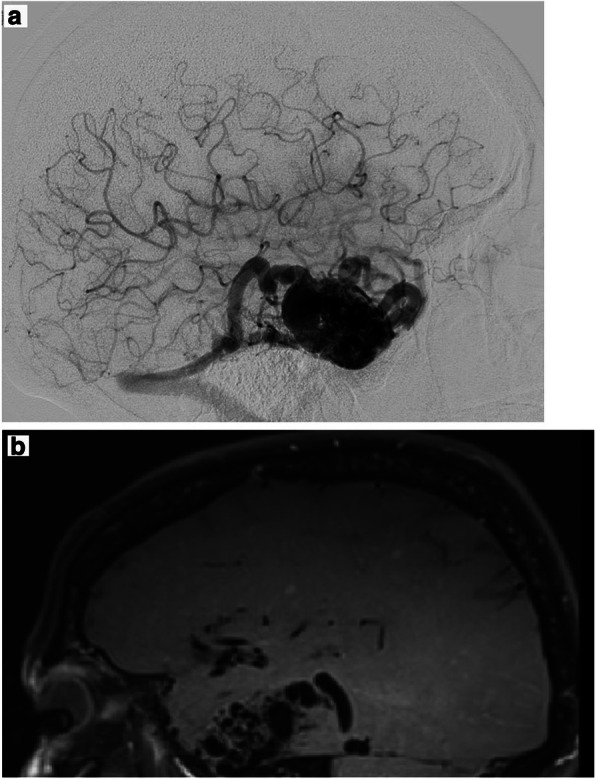
*Incidental AVM* **a** Digital subtraction angiography showing the AVM **b** BbMRI with contrast shows a moderate contrast enhancement of the nidus

The second case is that of a 53 y.o. female patient who presented with an epileptic seizure. The AVM (Fig. 2) was known for over 10 years but after the seizure and an increase in size we decided to remove the AVM. Before surgery the bbMRI was performed and showed an intranidal contrast enhancement of the vascular walls (Fig.2).

**Fig. 3:**
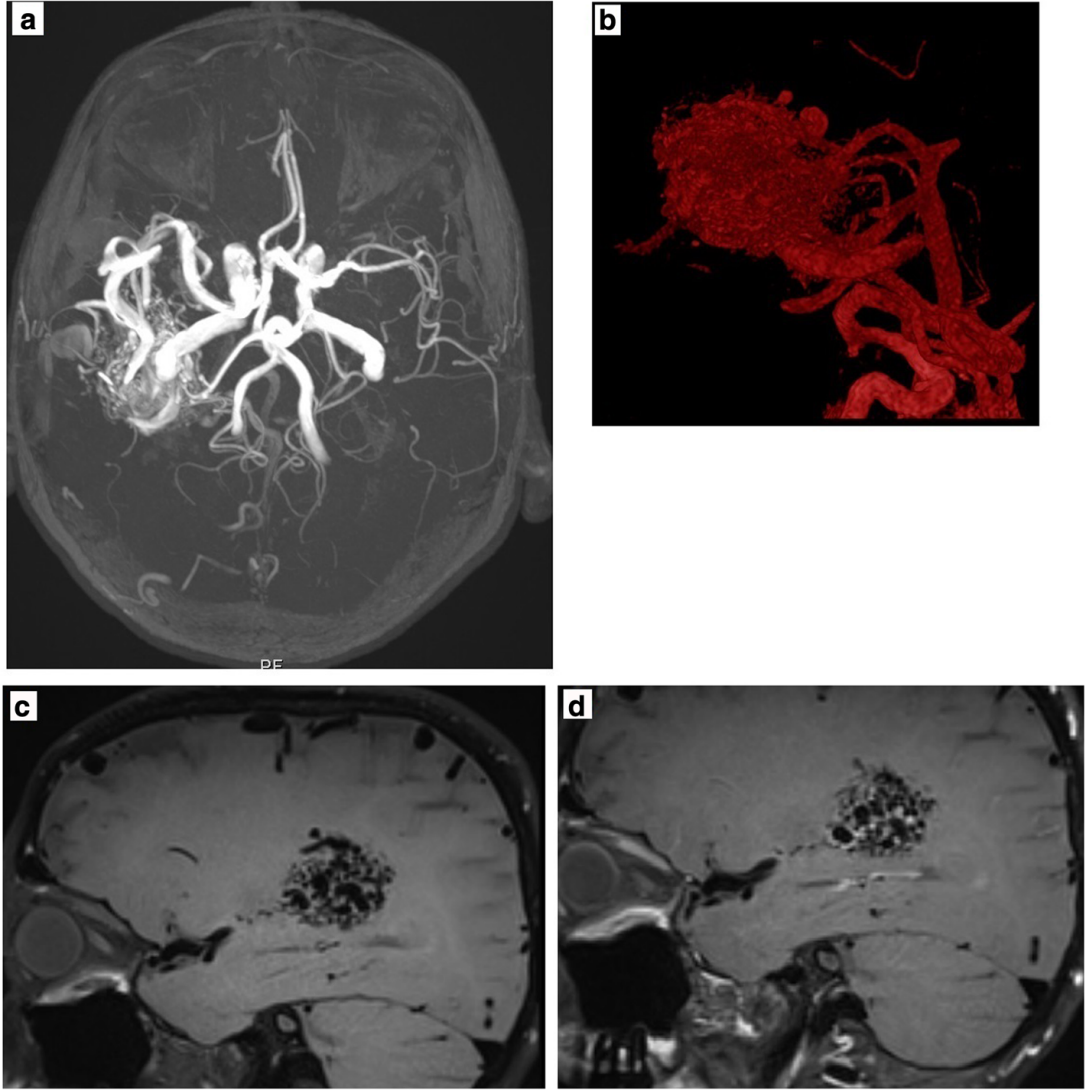
*Non-hemorrhaged temporoparietal AVM*. **a** MR-Angiography (left) and **b** reconstruction (right) of the AVM. **c** In the bbMRI (w/o contrast, left and **d** with contrast, right) there is a strong contrast enhancement of the AVM nidus

The third case is of 61 y.o. female with an epileptic seizure. The diagnostic MRI revealed a 46 mm temporo-parietal AVM. The bbMRI shows contrast enhancement of the nidus (Fig. 3). Under antiepileptic therapy she did not have any new seizures and because she rejected surgery we decided to observe the patient.

## Discussion

The present study evaluates feasibility of bbMRI for assessment of AVMs. It has been shown that inflammatory processes play a crucial role in AVM growth with increased expression of inflammatory cytokines and proteases in these lesions [7, 8]. Recruitment of leukocytes and leukocyte-derived metalloproteinases lead to structural degradation of the AVM walls which could lead to rupture [9]. On the other hand, macrophages and other inflammatory cells are detected in the vascular walls of AVMs which did not rupture [7, 8, 10]. Therefore, it cannot be concluded that the inflammation is a sign of AVM instability and a risk factor for rupture unlike in intracranial aneurysms [3, 4]. After demonstrating that bbMRI contrast enhancement is present in AVMs as well as in arteriovenous fistulas we looked for differences between ruptured and non-ruptured AVMs as well as size and inflammation. Although there is a strong uptake of contrast enhancement in the AVM nidus and in the abnormal vein of arteriovenous fistulas in the patients, indicating a high probability of ongoing inflammation there was no difference between ruptured and unruptured malformations in size. It can be concluded that there are dynamic inflammatory processes in AVMs and arteriovenous fistula walls but it seems that it is not more prevalent in AVMs prone to rupture. The observations of inflammation in AVM walls which could indicate a rupture risk have to be seen critically because unlike aneurysms, AVMs appear to be in a permanent stage of inflammation as seen in our study in which 6 /10 AVMs with no bleeding displayed strong contrast enhancement in bbMRI. The same phenomenon can be observed in the four cases of arteriovenous fistulas which were all contrast enhancing: there was no difference between the haemorrhaged (2 out of 4) and the non-haemorrhaged cases but sample sizes are of course low.

Never the less the number of patients studied does not allow drawing of conclusions other than that bbMRI is feasible in indicating inflammation in AVMs and that AVMs are highly active in terms of inflammatory processes. Based on this first observation with bbMRI further prospective and controlled studies looking for differences between stable AVMs and those prone to rupture are needed. Objective criteria of inflammatory activity could be of use in determining which AVMs should be treated and which are to be observed.

## Conclusions

The study is the first one using black blood MRI in AVMs. What we could show is an indication of a high inflammatory acticvity in the AVM wall speaking for a possible instability. Never the less and because of the small case number, it has to be evaluated in a prospective study if the inflammatory activity seen in the black blood MRI is associated with a higher bleeding rate. Histological specimens of AVMs together with black blood MRIs should be evaluated in further studies.

## Abbreviations

AVM: Arteriovenous Malformations
bbMRI: Black Blood Magnetic Resonance Imaging
ARUBA: A Randomised Trial of Unruptured Brain Arteriovenous Malformations.

## References

[1] Mohr JP, Parides MK, Stapf C, Moquete E, Moy CS, Overbey JR (2014). Medical management with or without interventional therapy for unruptured brain arteriovenous malformations (ARUBA): a multicenter, non-blinded, randomized trial. Lancet.

[2] Petridis AK, Fischer I, Cornelius JF, Kamp MA, Ringel F, Tortora A (2016). Demographic distribution of hospital admissions for brain arteriovenous malformations in Germany--estimation of the natural course with the big-data approach. Acta Neurochir (Wien)..

[3] Hu P, Yang Q, Wang D, Guan S, Zhang H (2016). Wall enhancement on high-resolution magnetic resonance imaging may predict an unsteady state of an intracranial saccular aneurysm. Neuroradiol.

[4] Park JK, Lee CS, Sim KB, Huh JS, Park JC (2009). Imaging of the walls of saccular cerebral aneurysms with double inversion recovery black.blood sequence. J Magn Res Imag.

[5] Zhang R, Han Z, Degos V, Shen F, Choi EJ, Sun Z (2016). Persistent infiltration and pro-inflammatory differentiation of monocytes cause unresolved inflammation in brain arteriovenous malformation. Angiogenesis.

[6] Zanaty M, Chalouhi N, Starke RM, Jabbour P, Hasan D (2016). Molecular Imaging in Neurovascular Diseases: The Use of Ferumoxytol to Assess Cerebral Aneurysms and Arteriovenous Malformations. Top Magn Reson Imaging.

[7] Chen Y, Fan Y, Poon KY, Achrol AS, Lawton MT, Zhu Y (2006). MMP-9 expression is associated with leukocytic but not endothelial markers in brain arteriovenous malformations. Front Biosci.

[8] Chen Y, Pawlikowska L, Yao JS, Shen F, Zhai W, Achrol AS (2006). Interleukin-6 involvement in brain arteriovenous malformations. Ann Neurol.

[9] Mun-Bryce S, Rosenberg GA (1998). Matrix metalloproteinases in cerebrovascular disease. J Cereb Blood Flow Metab.

[10] Chen Y, Zhu W, Bollen AW, Lawton MT, Barbaro NM, Dowd CF (2008). Evidence of inflammatory cell involvement in brain arteriovenous malformations. Neurosurgery.

